# Trends and determinants of perinatal mortality in Bangladesh

**DOI:** 10.1371/journal.pone.0221503

**Published:** 2019-08-23

**Authors:** Md. Belal Hossain, Sabuj Kanti Mistry, Md Mohsin, Md Hasinur Rahaman Khan

**Affiliations:** 1 BRAC James P Grant School of Public Health, BRAC University, Dhaka, Bangladesh; 2 Applied Statistics, Institute of Statistical Research and Training, University of Dhaka, Dhaka, Bangladesh; Public Health Foundation of India, INDIA

## Abstract

**Background:**

Although the perinatal mortality rate (PNMR) has been reduced over time in Bangladesh, the rate is still very high. Only a few studies explored the determinants of high PNMR in Bangladesh, yet most of them were small-scale or conducted for stillbirths and early neonatal deaths separately. The objective of this study was to explore the trends in and determinants of perinatal deaths in Bangladesh which would be an advanced step in effective policies to tackle the issue.

**Methods:**

The data used for this study was extracted from four rounds of Bangladesh Demographic and Health Surveys (BDHSs) 2004, 2007, 2011 and 2014. We considered the outcome of the 26604 pregnancies reaching seven months of their gestation. The trends of perinatal mortality was assessed using the Cochran–Armitage test, while the logistic regression with generalized estimating equation (GEE) to account for the clustering effect was implemented to explore the association between perinatal mortality and its risk factors.

**Results:**

The PNMR was significantly reduced from 64 (95% CI: 57–73) to 41 (95% CI: 35–48) per 1000 pregnancies between 2004 and 2014 (stillbirths: 34 to 19 and early neonatal deaths: 30 to 22). After adjusting for potential covariates in the model, we found that administrative division, type of cooking fuel, child’s gender, maternal occupation, body mass index, birth interval, history of miscarriage, previous deaths of children, total number of under 5 children, mode of delivery, type of delivery, access to participation in decision making, paternal education and occupation were significantly associated with perinatal deaths.

**Conclusion:**

The study highlights the importance of strengthening proper postnatal care services in the healthcare facilities. Alongside this, effort should also be stressed to ensure proper pregnancy care and to improve the socio-economic condition of the households to address the issue.

## Introduction

Perinatal mortality, which comprises of the total number of stillbirths and deaths within the first seven days of life [1], is the main contributor to infant mortality and is directly associated with maternal mortality [2]. Globally, 40% of infant mortality and 75% of neonatal mortality are occurring in perinatal period [3]. Worldwide, more than 7 million perinatal deaths occur each year which is higher than the total number of global deaths caused by AIDS (2.1 million), tuberculosis (1.6 million) and malaria (1.3 million) [2]. Notably, more than 8200 babies are stillborn each day in the world while 11000 die within the first week of their birth [1,4].

According to the most recent global estimate of World Health Organization (WHO), the perinatal mortality rate (PNMR) was 47 per thousand pregnancies in 2006 [1]. The PNMR was predominantly higher in African and Asian countries (Africa: 62, Asia: 50) and lower in Europe and America (Europe: 13, Latin America: 21, Northern America: 7). Similar to other South Asian counties, the PNMR is also high in Bangladesh [5]. The PNMR was 44 per 1000 pregnancies in 2014 in Bangladesh, which was nearly close to the overall under-five mortality rate in the country [6]. It is also evident that the pace of reduction in under-five child mortality rate is notably low in Bangladesh [7]. Thus, with the current pace of reduction, Bangladesh is less likely to achieve the United Nations (UN) Sustainable Development Goal 3 (SDG 3) of reducing under-five mortality rate up to 25 per 1000 live births [8].

In spite of a high PNMR in Bangladesh, only a few studies have been carried out till date focusing on this issue and most of them were small-scale clinically oriented and conducted for stillbirths and early neonatal deaths separately [9,10]. Although these studies identified a number of risk factors associated with perinatal deaths, none of these studies highlighted the trends in PNMR and did not include a number of covariates which might be potentially associated with high perinatal deaths in Bangladesh. Therefore, large-scale household based data is very important to track the nationwide trends in PNMR as well as to identify the most potent risk factors in Bangladesh context.

Bangladesh demographic and health surveys (BDHSs) are naturally hierarchical and aimed to collect nationwide household-based mortality data. The main strength of using BDHS data to report perinatal mortality is the high quality of data and large number of covariates they include [7,11–13]. Given the context of high perinatal deaths in Bangladesh, using BDHS datasets, the present study was aimed to identify the trends in PNMR. The study was also carried out to identify the potential socio-economic, demographic and parental risk factors that are associated with the perinatal deaths in Bangladesh.

## Methods

### Data source

The study used data from four rounds of Bangladesh Demographic and Health Surveys (BDHSs): 2004, 2007, 2011 and 2014 [7,11–13] which covered all districts and administrative divisions of Bangladesh. These nationally representative surveys were cross-sectional in nature and followed a two-stage stratified random sampling of households. We pooled these datasets for the present analysis. The detailed methodology can be found in the BDHS reports [7,11–13].

**Fig 1:**
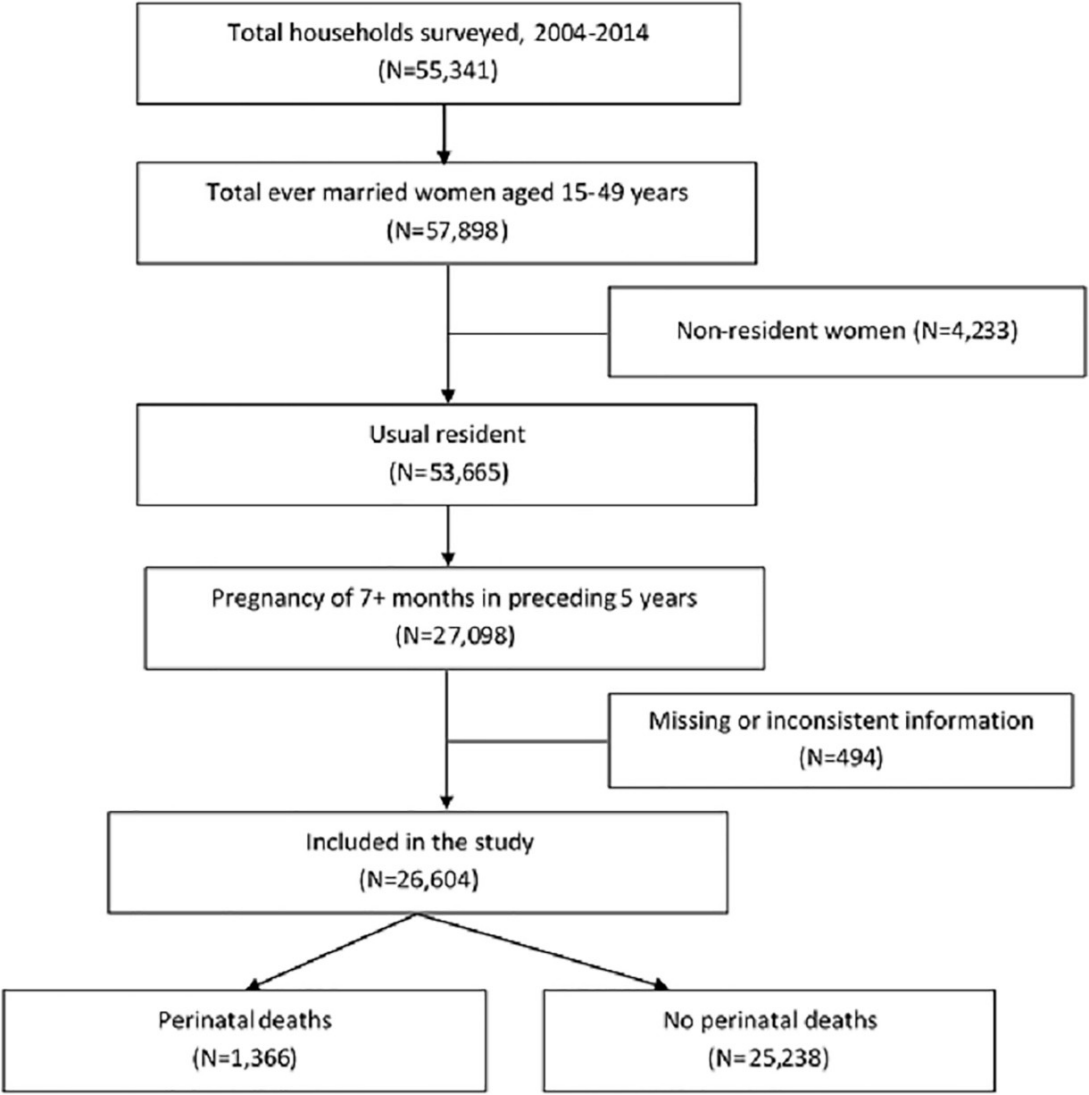
Study profile and participants’ enrollment.

The 2004, 2007, 2011 and 2014 BDHSs collected information from 11290, 10996, 17749 and 17863 ever married 15–49 years aged women respectively. The response rate of each of the surveys was approximately 98%. Among these 57898 women, 53665 were usual resident of the selected households (de jure population). BDHSs usually collected information of those mothers and their children who were born (or stillbirths) within the preceding five years from the survey years. For the present analysis, we considered information of 27098 such pregnancies reaching seven months of gestation, i.e., the stillbirths and live births. Finally, 26604 women were included for the final analysis, while 494 women were dropped due to missing or inconsistent information (Fig 1).

### Study definitions

All ever-married 15–49 years aged women were asked to provide the history of all of the pregnancies in the preceding five years including a complete history of their live births, sex of the child, month and year of each birth, survival status, and age at the time of the survey or age at death. Information on the deaths those caused by pregnancy losses occurring after seven completed months of gestation (i.e., stillbirths) such as the status of any stillbirths preceding five years (yes/no) and sex of those children were collected using the calendar at the end of the woman’s questionnaire. Moreover, anthropometric measurements of every ever-married 15–49 years aged women and children aged below 5 years were undertaken.

### Data analysis

#### Outcome variable

The perinatal mortality rate (PNMR) was the outcome variable and calculated through dividing the total number of perinatal deaths by the total number of pregnancies reaching seven months of gestation, and expressed per 1,000. Thus, PNMR is defined as:

$$PNMR=\frac{Number\;of\;stillbirths+Number\;of\;early\;neonatal\;deaths}{Number\;of\;stillbirths+Number\;of\;live\;births}\times 1,000,$$

where stillbirths were defined as the babies born dead after seven completed months of gestation or fetal deaths, and early neonatal deaths were defined as the deaths occurred within the first seven days of life [1]. The outcome variable for the study was recorded as a binary variable in the datasets, recoded as ‘1’ for perinatal mortality and ‘0’ for no perinatal mortality.

#### Independent variables

The factors included in the study were household, child and parental factors. The household factor includes administrative division, place of residence, wealth quintile, types of cooking fuel, sources of drinking water and types of latrine. The wealth quintile was constructed through factor analysis[14]. Cooking fuel was categorized into solid and non-solid fuels [10]. Sources of drinking water and types of latrine were categorized into improved and unimproved based on the report of WHO-UNICEF Joint Monitoring Programme [15] for the year 2007–2014, and WHO and UNICEF [16] for 2004.

Gender was the only independent factors relevant to the child while other child factors were aligned with maternal factors. For instance, due to the high collinearity between child’s birth order and maternal birth rank, these variables were merged into one variable. Other maternal factors were age at child’s birth, level of education, occupation, religion, body mass index (BMI), exposure to media (i.e., watching television, listening to radio, reading newspaper), miscarriage and/or abortion history, previous death of any child, number of under 5 children, number of antenatal care visits, place and mode of delivery, type of delivery and access to participation in decision-making. Access to participation in decision-making was defined as access to take decision alone or jointly with husband on all of the four components: own health care, major household purchases, child health care, and visits to her family or relatives [6]. The BMI was categorized as underweight (BMI<18.5 kg/m^2^), normal (BMI: 18.5–24.9 kg/m^2^), overweight (BMI: 25.0–29.9 kg/m^2^) and obese (BMI≥30.0 kg/m^2^). Paternal factors considered were level of education and occupation. Detailed information on the assessed and measured study variables can be found in BDHS reports 2004–2014 [7,11–13].

#### Statistical analysis

Descriptive analysis was performed to assess the distribution of the variables. The Cochran–Armitage test [17,18] was performed to assess the trend of perinatal mortality, and the Chi-square test was used for comparing the mortality rate among different categories of a variable. To ensure actual representation of the nationwide data, sampling weights were used in these cases. Since the data were nested in nature and there were variations among clusters (enumeration areas), we executed logistic regression model with generalized estimating equation (GEE) to account for the clustering effect [19]. In this case, we considered the exchangeable correlation structure between clusters [20]. The variables with *P* < 0.25 in the unadjusted analyses were only included in the final model [21]. The odds ratio (OR) and confidence interval (CI) were estimated by considering the 5% level of significance. All analyses were performed using statistical software package Stata (Version 13.0) and R (Version 3.4.4).

### Ethics approval and consent to participate

BDHS data are public access data and were made available to us by MEASURE DHS upon request. While conducting survey, an informed consent was obtained from the respondents prior to the interview. Ethical clearance to conduct the BDHS was approved by the Government of Bangladesh.

## Results

### Background characteristics

Nearly one-third of the children were from Dhaka division, while more than two-thirds were hailed from rural areas and 24.5% were from the poorest households (Table 1). The ratio of boys to girls was near unity. Also, 26.3% of the mothers and 33.7% of the fathers had no education. Majority of the mothers were the homemaker, while 44.5% of the fathers were the blue-collar worker. A total of 29.5% of the mothers were underweight, 9.4% were overweight and only 1.7% were obese.

**Table 1:** Background characteristics of the study population.

Characteristics			n	%
	Survey year			
		2004	6272	23.6
		2007	5335	20.0
		2011	7706	29.0
		2014	7291	27.4
**Household characteristics**				
	Administrative division			
		Barisal	1522	5.7
		Chittagong	5811	21.8
		Dhaka	8564	32.2
		Khulna	2438	9.2
		Rajshahi	4282	16.1
		Rangpur	1575	5.9
		Sylhet	2412	9.1
	Place of residence			
		Rural	20602	77.4
		Urban	6002	22.6
	Household wealth quintile			
		Poorest	6508	24.5
		Poorer	5473	20.6
		Middle	5053	19.0
		Richer	4932	18.5
		Richest	4637	17.4
**Child characteristics**				
	Gender			
		Male	13627	51.2
		Female	12977	48.8
**Maternal characteristics**				
	Age at child's birth (years)			
		<20	8268	31.1
		20–29	14210	53.4
		30–39	3822	14.4
		≥40	305	1.1
	Level of education			
		No education	7004	26.3
		Primary incomplete	5214	19.6
		Primary complete^1^	2888	10.9
		Secondary incomplete	8491	31.9
		Secondary complete or higher^2^	3008	11.3
	Occupation			
		Homemaker	21096	79.3
		Employed	5508	20.7
	Body mass index (kg/m^2^)			
		<18.5	7854	29.5
		18.5–24.9	15800	59.4
		25–29.9	2491	9.4
		≥30	459	1.7
**Paternal characteristics**				
	Level of education			
		No education	8968	33.7
		Primary incomplete	4710	17.7
		Primary complete^1^	3065	11.5
		Secondary incomplete	5619	21.1
		Secondary complete or higher^2^	4241	15.9
	Occupation			
		Agriculture	7224	27.2
		White-collar^3^	1088	4.1
		Blue-collar^4^	11844	44.5
		Pink-collar^5^	5728	21.5
		Others^6^	721	2.7
**N**			26604	100.00

^1^Primary complete is defined as completing grade 5

^2^Secondary complete is defined as completing grade 10

^3^Doctor, lawyer, dentist, accountant, teacher, nurse, family welfare visitor

^4^Non-agricultural worker, carpenter, mason, bus/taxi driver, construction supervisor, tailor, rickshaw driver, brick breaking, road building, construction worker, boatman, fisherman, poultry raising, cattle raising

^5^Businessman/trader

^6^Unemployed/student, retired, beggar, etc.

### Trends in perinatal mortality

**Fig 2:**
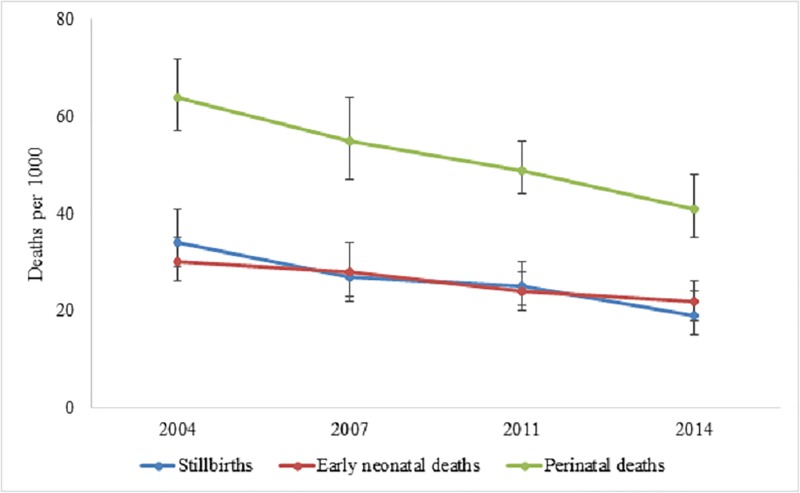
Trends of perinatal mortality per 1000 pregnancies of seven or more months’ duration in Bangladesh, 2004–2014.

The perinatal mortality rate (PNMR) per 1,000 pregnancies of seven or more months was reduced from 64 in 2004 to 41 in 2014 (Table 2). In contrast, the stillbirths were reduced from 34 to 19, while the early neonatal deaths were reduced from 30 to 22 during the period of 2004 to 2014 (Fig 2). The trend of reduction in PNMR was statistically significant (*P*<0.001, Table 2). A significant reduction was also observed among both boys and girls (*P*<0.001). Administratively, the PNMR was declining for all divisions except Sylhet. In 2004, the PNMR was 70 in Sylhet, which was increased to 75 in 2014. The reduction rate was also significant among children from both rural and urban areas (rural: 65 to 44, *P*<0.001; urban: 63 to 31, *P*<0.001). Although the rate was gradually declined among children from middle or rich households, it remained considerably higher among children from poor households (poorest: 54, poorer: 51 both in 2014). However, a considerable increase of PNMR was seen among children whose mother had a history of previous miscarriage or abortion, the death of any child, and those of twin deliveries.

**Table 2:** Trends of and pooled perinatal mortality rate in Bangladesh between 2004 and 2014.

Characteristics			n^1^	PNMR (95% CI)				% reduced^2^	*P*^3^	Pooled PNMR (95% CI)	*P*^4^
				2004	2007	2011	2014				
**Household characteristics**											
	Administrative division										
		Barisal	1522	44 (30–64)	68 (49–94)	56 (40–79)	33 (21–53)	25.0	0.346	50 (41–61)	<0.001
		Chittagong	5811	62 (46–84)	40 (28–56)	28 (21–39)	32 (22–45)	48.4	<0.001	39 (33–46)	
		Dhaka	8564	58 (46–73)	54 (40–72)	52 (40–66)	35 (24–50)	39.7	<0.001	49 (42–56)	
		Khulna	2438	61 (41–90)	45 (31–66)	56 (40–79)	48 (31–72)	21.3	0.476	53 (44–65)	
		Rajshahi	4282	79 (64–98)	65 (46–92)	61 (47–79)	48 (33–68)	39.2	0.005	66 (57–76)	
		Rangpur^5^	1575	-	-	46 (35–62)	37 (24–58)	19.6	0.341	42 (32–55)	
		Sylhet	2412	70 (54–91)	63 (42–92)	64 (47–85)	75 (56–100)	-7.1	0.704	69 (59–80)	
	Place of residence										
		Rural	20602	65 (56–74)	57 (48–68)	49 (42–56)	44 (37–53)	32.3	<0.001	53 (49–57)	0.036
		Urban	6002	63 (51–79)	43 (32–56)	49 (38–63)	31 (23–41)	50.8	<0.001	45 (40–51)	
	Household wealth quintile										
		Poorest	6508	65 (51–84)	54 (39–74)	48 (38–60)	54 (40–72)	16.9	0.094	55 (48–63)	0.008
		Poorer	5473	63 (50–79)	58 (42–79)	56 (44–70)	51 (38–69)	19.0	0.223	57 (50–65)	
		Middle	5053	71 (55–90)	71 (52–95)	50 (37–68)	40 (28–58)	43.7	<0.001	57 (49–66)	
		Richer	4932	62 (40–95)	53 (39–71)	47 (36–62)	28 (20–40)	54.8	<0.001	46 (38–55)	
		Richest	4637	60 (46–77)	34 (23–50)	41 (30–56)	28 (19–40)	53.3	<0.001	40 (34–47)	
	Type of cooking fuel										
		Non-solid	2947	59 (41–83)	27 (15–47)	38 (27–55)	23 (14–37)	61.0	0.003	34 (27–42)	<0.001
		Solid	23657	65 (57–74)	57 (48–66)	50 (44–57)	44 (37–52)	32.3	<0.001	54 (50–57)	
	Source of drinking water										
		Unimproved	745	60 (36–98)	60 (34–103)	70 (33–143)	68 (44–102)	-13.3	0.619	64 (48–84)	0.128
		Improved	25859	65 (57–73)	54 (46–63)	48 (43–55)	40 (34–47)	38.5	<0.001	51 (48–55)	
	Type of toilet facility										
		Unimproved	12587	65 (55–77)	56 (47–68)	54 (46–63)	51 (40–65)	21.5	0.022	57 (52–62)	0.007
		Improved	14017	64 (53–76)	51 (39–65)	43 (36–52)	35 (29–43)	45.3	<0.001	47 (42–52)	
**Child characteristics**											
	Gender										
		Male	13627	63 (54–74)	56 (47–67)	54 (46–63)	42 (34–53)	33.3	<0.001	53 (49–58)	0.208
		Female	12977	66 (56–77)	52 (42–65)	43 (36–51)	39 (31–49)	40.9	<0.001	49 (45–54)	
**Maternal characteristics**											
	Age at child's birth (years)										
		<20	8268	74 (61–90)	65 (53–79)	60 (50–72)	42 (33–54)	43.2	<0.001	60 (54–66)	0.007
		20–29	14210	59 (49–71)	55 (44–68)	45 (38–53)	39 (32–49)	33.9	<0.001	48 (44–54)	
		30–39	3822	61 (45–82)	32 (22–47)	38 (27–54)	43 (27–67)	29.5	0.114	44 (36–53)	
		≥40	305	70 (29–161)	44 (9–180)	52 (16–152)	41 (12–131)	41.4	0.539	54 (31–93)	
	Level of education										
		No education	7004	73 (61–88)	60 (45–81)	42 (32–56)	41 (28–59)	43.8	<0.001	57 (50–65)	0.025
		Primary incomplete	5214	54 (39–74)	49 (37–65)	54 (42–69)	50 (35–72)	7.4	0.786	52 (44–61)	
		Primary complete^6^	2888	65 (44–94)	81 (56–115)	45 (32–63)	56 (39–79)	13.8	0.091	58 (49–70)	
		Secondary incomplete	8491	61 (47–78)	49 (38–63)	52 (42–63)	38 (30–49)	37.7	0.002	48 (43–54)	
		Secondary complete or higher^7^	3008	56 (35–67)	40 (25–63)	48 (33–69)	26 (17–39)	53.6	0.012	39 (32–49)	
	Occupation										
		Homemaker	21096	62 (54–71)	56 (47–66)	46 (40–52)	41 (34–49)	33.9	<0.001	50 (47–54)	0.228
		Employed	5508	73 (58–92)	51 (39–67)	75 (57–99)	40 (30–54)	45.2	0.002	55 (48–63)	
	Religion										
		Islam	24378	65 (57–73)	55 (47–64)	49 (43–55)	42 (35–49)	35.4	<0.001	52 (48–56)	0.335
		Others^8^	2226	60 (41–87)	50 (26–95)	49 (33–71)	30 (16–54)	50.0	0.017	46 (36–58)	
	Body mass index (kg/m^2^)										
		<18.5	7854	62 (50–77)	67 (54–83)	42 (33–54)	38 (26–54)	38.7	<0.001	53 (47–60)	0.256
		18.5–24.9	15800	66 (57–77)	49 (40–59)	52 (44–60)	44 (35–54)	33.3	<0.001	52 (48–57)	
		25–29.9	2491	60 (33–107)	46 (24–86)	44 (30–64)	32 (23–44)	46.7	0.028	41 (32–51)	
		≥30	459	70 (23–198)	40 (15–103)	77 (36–156)	49 (19–119)	30.0	0.608	59 (36–96)	
	Exposure to media^9^										
		No	10215	67 (55–81)	57 (45–73)	49 (40–58)	56 (44–70)	16.4	0.041	56 (51–63)	0.019
		Yes	16389	63 (54–73)	52 (44–62)	49 (42–57)	31 (25–38)	50.8	<0.001	48 (44–52)	
	Birth order and birth interval										
		First birth	8607	97 (81–115)	75 (62–91)	73 (62–86)	51 (41–62)	47.4	<0.001	71 (65–78)	<0.001
		2nd birth, <2 years interval	979	71 (44–114)	61 (35–106)	84 (54–128)	37 (16–81)	47.9	0.301	64 (49–83)	
		3rd/later birth, <2 years interval	1404	54 (36–81)	89 (55–141)	34 (19–63)	40 (19–83)	25.9	0.093	55 (42–72)	
		2nd birth, ≥2 years interval	6379	49 (37–64)	42 (29–60)	37 (28–49)	33 (24–44)	32.7	0.016	39 (34–45)	
		3rd/later birth, ≥2 years interval	9235	52 (43–63)	37 (27–50)	31 (25–40)	36 (27–49)	30.8	0.001	39 (35–45)	
	Previously pregnancies miscarried or aborted										
		No	24615	67 (59–76)	47 (40–55)	43 (38–50)	37 (32–44)	44.8	<0.001	48 (45–52)	<0.001
		Yes	1989	33 (19–55)	144 (104–196)	117 (85–159)	87 (61–122)	-163.6	<0.001	92 (77–110)	
	Previous death of any child										
		No	25239	57 (50–66)	46 (39–54)	45 (39–51)	37 (31–44)	35.1	<0.001	46 (42–49)	<0.001
		Yes	1365	139 (107–180)	171 (118–240)	150 (103–213)	213 (131–329)	-53.2	0.077	159 (133–188)	
	Number of children in last five years										
		1	17597	51 (44–60)	44 (37–53)	38 (33–45)	32 (27–39)	37.3	<0.001	40 (37–44)	<0.001
		2	8031	73 (60–89)	56 (43–72)	68 (55–84)	57 (45–73)	21.9	0.153	65 (58–73)	
		3+	977	135 (93–192)	180 (118–265)	109 (71–164)	140 (75–246)	-3.7	0.611	142 (111–180)	
	Number of antenatal care visits										
		<4	22201	65 (57–73)	54 (46–64)	50 (44–57)	42 (36–49)	35.4	<0.001	52 (49–56)	0.119
		4+	4403	61 (44–82)	53 (38–74)	44 (34–57)	35 (25–50)	42.6	0.005	46 (39–54)	
	Place and mode of delivery										
		Home	18823	61 (54–70)	53 (45–63)	43 (37–50)	39 (30–51)	36.1	<0.001	50 (47–55)	0.198
		Health facilities without C-section	5047	99 (67–142)	62 (42–89)	57 (41–78)	42 (34–52)	57.6	<0.001	50 (43–58)	
		Health facilities with C-section	2734	91 (60–136)	57 (35–89)	75 (58–97)	41 (29–56)	54.9	0.006	60 (50–71)	
	Type of delivery										
		Single birth	26188	63 (56–71)	52 (45–61)	47 (41–53)	38 (32–45)	39.7	<0.001	49 (46–53)	<0.001
		Twin	416	139 (68–262)	181 (90–332)	156 (89–260)	221 (120–372)	-59.0	0.181	173 (125–233)	
	Access to participation in decision-making^10^										
		No	16761	66 (57–76)	58 (49–70)	55 (47–63)	47 (39–57)	28.8	<0.001	57 (52–61)	<0.001
		Yes	9659	61 (48–76)	47 (36–61)	40 (32–48)	33 (25–42)	45.9	<0.001	42 (38–47)	
**Paternal characteristics**											
	Level of education										
		No education	8968	75 (62–89)	61 (48–78)	49 (39–60)	47 (35–63)	37.3	<0.001	59 (52–66)	0.005
		Primary incomplete	4710	53 (39–72)	53 (38–73)	54 (41–71)	44 (31–62)	17.0	0.341	51 (44–59)	
		Primary complete^6^	3065	67 (48–93)	61 (40–92)	43 (30–62)	43 (28–66)	35.8	0.012	51 (42–62)	
		Secondary incomplete	5619	64 (47–87)	51 (38–68)	49 (38–62)	41 (31–54)	35.9	0.007	50 (43–58)	
		Secondary complete or higher^7^	4241	43 (29–61)	40 (27–59)	48 (34–66)	27 (18–41)	37.2	0.123	39 (32–47)	
	Occupation										
		Agriculture	7224	81 (66–100)	52 (37–74)	52 (41–65)	51 (39–67)	37.0	<0.001	59 (52–67)	<0.001
		White-collar^11^	1088	45 (19–104)	34 (15–75)	44 (25–76)	33 (19–56)	26.7	0.595	38 (27–53)	
		Blue-collar^12^	11844	61 (51–72)	66 (54–81)	53 (44–63)	40 (31–52)	34.4	<0.001	54 (49–60)	
		Pink-collar^13^	5728	54 (40–74)	39 (28–53)	39 (29–51)	32 (23–45)	40.7	0.005	41 (35–48)	
		Others^14^	721	37 (14–90)	11 (2–49)	32 (14–69)	38 (16–87)	-2.7	0.504	31 (19–49)	
Overall			26604	64 (57–73)	54 (47–63)	49 (43–55)	41 (35–48)	35.9	<0.001	51 (48–55)	

^1^Number of pregnancies of seven or more months’ duration

^2^% reduction from the year 2004 to 2014

^3^*P*-value for the Cochran–Armitage test of % of PNMR reduction from the year 2004 to 2014

^4^*P*-value for the Chi-square test of pooled PNMR

^5^The administrative division Rangpur was created in 2010

^6^Primary complete is defined as completing grade 5

^7^Secondary complete is defined as completing grade 10

^8^Others = Hindu, Christian, Buddhist, etcetera

^9^Exposure to media = Watches TV/listens to radio/reads newspaper in the previous week

^10^Access to participation in decision-making = having access to take decision alone or jointly with husband on all of the four components: own health care, major household purchases, child health care, and visits to her family or relatives

^11^Doctor, lawyer, dentist, accountant, teacher, nurse, family welfare visitor

^12^Non-agricultural worker, carpenter, mason, bus/taxi driver, construction supervisor, tailor, rickshaw driver, brick breaking, road building, construction worker, boatman, fisherman, poultry raising, cattle raising

^13^Businessman/trader

^14^Unemployed/student, retired, beggar, etcetera.

### Pooled perinatal mortality rate

The pooled perinatal mortality rate per 1,000 pregnancies of seven or more months was 51 (95% CI: 48–55) (Table 2). Discrepancies in PNMR were observed among administrative divisions. The PNMR was lowest in Chittagong and highest in Sylhet (40 and 69 respectively, *P*<0.001). The rate was significantly higher in rural areas than urban (53 versus 45, *P* = 0.036), and more pronounced among children from poor households (*P* = 0.008). The PNMR was also higher among children from the households using solid cooking fuel, drinking unimproved water and having unimproved toilet facilities. Meanwhile, the PNMR was higher among boys than girls (53 versus 49, *P* = 0.208). The PNMR was significantly higher among children from mothers aged <20 years and ≥40 years (60 and 54 respectively). The rate gradually declined as parental level of education increased. The PNMR was also higher among children whose mothers were obese, had no exposure to media, were pregnant for the first time or in second pregnancy within 2 years, ever experienced miscarriage or abortion, previously had any child death, had more than one children born in the last five years, had <4 antenatal care visits, underwent health facilities with C-section, experienced twin babies, and had no access to household decision-making. Moreover, the PNMR was higher among children whose father engaged in agriculture-based works.

### Determinants of perinatal mortality

The individual assessment of each covariate of the pooled data was presented in Table 3. The adjusted estimated effects for the determinants associated with perinatal mortality in Bangladesh were also shown in Table 3. After adjusting with survey year and cluster variation, administrative division, type of cooking fuel, child’s gender, maternal occupation, BMI of mothers, age at first marriage, birth order and birth interval, ever experienced miscarriage or abortion, previously having any child death, total under 5 children in the last five years, place and mode of delivery, type of delivery, own decision-making status, and paternal education and occupation remained as independent determinants for perinatal mortality.

**Table 3:** Association of household, child and parental characteristics with perinatal mortality in Bangladesh.

Characteristics			Unadjusted			Adjusted		
			OR	95% CI	*P*	OR	95% CI	*P*
	Survey year							
		2004	1.00			1.00		
		2007	0.86	0.72–1.03	0.100	0.86	0.72–1.03	0.105
		2011	0.78	0.66–0.92	0.003	0.86	0.72–1.02	0.088
		2014	0.65	0.55–0.77	<0.001	0.65	0.53–0.80	<0.001
**Household characteristics**								
	Administrative division							
		Barisal	0.98	0.78–1.23	0.857	1.01	0.79–1.28	0.960
		Chittagong	0.77	0.62–0.95	0.016	0.72	0.58–0.90	0.003
		Dhaka	1.00			1.00		
		Khulna	1.02	0.82–1.28	0.857	0.95	0.75–1.20	0.651
		Rajshahi	1.22	1.00–1.50	0.052	1.08	0.88–1.34	0.450
		Rangpur	0.91	0.69–1.19	0.484	0.95	0.71–1.27	0.730
		Sylhet	1.35	1.11–1.64	0.003	1.15	0.94–1.42	0.182
	Place of residence							
		Rural	1.10	0.96–1.25	0.170	0.97	0.83–1.13	0.683
		Urban	1.00			1.00		
	Household wealth quintile							
		Poorest	1.37	1.14–1.64	0.001	1.07	0.81–1.40	0.639
		Poorer	1.50	1.25–1.80	<0.001	1.19	0.92–1.53	0.187
		Middle	1.40	1.16–1.69	<0.001	1.17	0.92–1.49	0.201
		Richer	1.17	0.97–1.42	0.105	1.06	0.85–1.32	0.607
		Richest	1.00			1.00		
	Type of cooking fuel							
		Non-solid	1.00			1.00		
		Solid	1.48	1.20–1.82	<0.001	1.30	1.00–1.70	0.051
	Source of drinking water							
		Unimproved	1.00			1.00		
		Improved	0.79	0.59–1.06	0.110	0.94	0.69–1.26	0.664
	Type of toilet facility							
		Unimproved	1.00			1.00		
		Improved	0.83	0.74–0.93	0.001	0.97	0.85–1.11	0.657
**Child characteristics**								
	Gender		1.00			1.00		
		Male	1.10	0.98–1.22	0.094	1.11	1.00–1.24	0.059
		Female						
**Maternal characteristics**								
	Age at child's birth (years)							
		<20	1.17	0.71–1.93	0.544	Not taken in the final model		
		20–29	0.93	0.56–1.53	0.772			
		30–39	0.86	0.52–1.45	0.581			
		≥40	1.00					
	Level of education							
		No education	1.00			1.00		
		Primary incomplete	0.95	0.81–1.11	0.525	0.99	0.83–1.17	0.865
		Primary complete^1^	1.05	0.87–1.26	0.629	1.09	0.88–1.33	0.438
		Secondary incomplete	0.85	0.73–0.98	0.026	0.91	0.75–1.10	0.326
		Secondary complete or higher^2^	0.67	0.55–0.83	<0.001	0.81	0.61–1.08	0.154
	Occupation							
		Homemaker	1.00			1.00		
		Employed	1.09	0.95–1.25	0.219	1.19	1.03–1.38	0.016
	Religion							
		Islam	1.09	0.89–1.34	0.395	Not taken in the final model		
		Others^3^	1.00					
	Body mass index (kg/m^2^)							
		<18.5	0.98	0.87–1.11	0.804	0.88	0.78–1.00	0.058
		18.5–24.9	1.00			1.00		
		25–29.9	0.87	0.72–1.06	0.172	1.20	0.97–1.48	0.090
		≥30	1.09	0.74–1.60	0.660	1.57	1.04–2.37	0.032
	Exposure to media^4^							
	No		1.00			1.00		
	Yes		0.92	0.82–1.04	0.173	1.02	0.89–1.17	0.739
	Birth order and birth interval							
	First birth		1.00			1.00		
	2nd birth, <2 years interval		0.86	0.65–1.13	0.269	0.53	0.40–0.70	<0.001
	3rd/later birth, <2 years interval		0.69	0.54–0.88	0.003	0.25	0.19–0.33	<0.001
	2nd birth, ≥2 years interval		0.52	0.45–0.61	<0.001	0.49	0.42–0.57	<0.001
	3rd/later birth, ≥2 years interval		0.57	0.50–0.65	<0.001	0.35	0.30–0.41	<0.001
	Previously pregnancies miscarried or aborted							
		No	1.00			1.00		
		Yes	1.96	1.67–2.30	<0.001	2.06	1.74–2.43	<0.001
	Previous death of any child							
		No	1.00			1.00		
		Yes	4.06	3.48–4.74	<0.001	4.36	3.61–5.26	<0.001
	Number of children in last five years							
		1	1.00			1.00		
		2	1.67	1.49–1.88	<0.001	1.71	1.50–1.95	<0.001
		3+	3.72	3.06–4.53	<0.001	2.83	2.23–3.61	<0.001
	Number of antenatal care visits							
		<4	1.00			1.00		
		4+	0.79	0.68–0.93	0.003	1.01	0.85–1.21	0.890
	Place and mode of delivery							
		Home	1.00			1.00		
		Health facilities without C-section	1.03	0.89–1.19	0.670	1.29	1.09–1.53	0.004
		Health facilities with C-section	1.18	0.99–1.40	0.059	1.55	1.26–1.90	<0.001
	Type of delivery							
		Single birth	1.00			1.00		
		Twin	4.16	3.25–5.33	<0.001	1.97	1.48–2.63	<0.001
	Access to participation in decision-making^5^							
		No	1.00			1.00		
		Yes	0.80	0.71–0.90	<0.001	0.89	0.78–1.00	0.056
**Paternal characteristics**								
	Level of education							
		No education	1.00			1.00		
		Primary incomplete	0.91	0.78–1.07	0.253	0.92	0.78–1.09	0.324
		Primary complete^1^	0.85	0.70–1.02	0.074	0.85	0.70–1.04	0.122
		Secondary incomplete	0.80	0.69–0.93	0.004	0.84	0.70–1.00	0.054
		Secondary complete or higher^2^	0.60	0.50–0.72	<0.001	0.69	0.54–0.89	0.004
	Occupation							
		Agriculture	1.00			1.00		
		White-collar^6^	0.57	0.41–0.79	0.001	0.78	0.54–1.14	0.197
		Blue-collar^7^	0.94	0.82–1.07	0.338	0.98	0.85–1.13	0.806
		Pink-collar^8^	0.71	0.60–0.84	<0.001	0.79	0.66–0.95	0.011
		Others^9^	0.53	0.34–0.82	0.004	0.54	0.35–0.85	0.007

^1^Primary complete is defined as completing grade 5

^2^Secondary complete is defined as completing grade 10

^3^Others = Hindu, Christian, Buddhist, etcetera

^4^Exposure to media = Watches TV/listens to radio/reads newspaper in the previous week

^5^Access to participation in decision-making = having access to take decision alone or jointly with husband on all of the four components: own health care, major household purchases, child health care, and visits to her family or relatives

^6^Doctor, lawyer, dentist, accountant, teacher, nurse, family welfare visitor

^7^Non-agricultural worker, carpenter, mason, bus/taxi driver, construction supervisor, tailor, rickshaw driver, brick breaking, road building, construction worker, boatman, fisherman, poultry raising, cattle raising

^8^Businessman/trader

^9^Unemployed/student, retired, beggar, etcetera.

The odds of perinatal mortality was declined by 35% between 2004 and 2014 (aOR: 0.65, 95% CI: 0.53–0.80). Children from Chittagong division had 28% less odds of perinatal mortality than those from Dhaka division. Moreover, children from the households using solid cooking fuel had 30% higher odds of perinatal mortality than those using non-solid fuel. Meanwhile, the odds of perinatal mortality was 11% higher among boys than girls.

The odds of perinatal mortality was 19% higher among children whose mothers were working outside compared to those whose mothers were the homemaker. The odds was also 57% higher among children whose mothers were obese. It was also found that the odds was approximately twice when the mothers had a previous history of miscarriage or abortion, while the odds was more than four times when the mothers had the history of any child death. The odds of perinatal mortality was 29% higher for children delivered in health facilities without C-section than delivered in the home, while it was 55% higher for children delivered in health facilities with C-section than delivered in the home. Moreover, the odds of perinatal mortality was approximately twice among twin babies in comparison with the single births. Children of the fathers who had completed secondary or higher education exhibited a 31% lower odds of perinatal mortality than the fathers with no education. Besides, the odds of perinatal mortality was 21% lower among children whose fathers were businessmen/traders in comparison with those involved in agriculture-based livelihoods (Table 3).

## Discussion

The study identified a positive trend in reduction of PNMR in Bangladesh, yet the rate is still high. We found a 4.4% annual reduction in stillbirth rate while early neonatal mortality rate (ENMR) was reduced only by 2.7%. This slower reduction in ENMR may possibly be resulted due to lack of quality in neonatal care as well as lack of large scale effective interventions addressing the early neonatal deaths. It is also important to note that, of the 28 neonatal deaths per thousand live births in Bangladesh, 22 deaths occurred during the early neonatal period [6]. In contrast, although the rate of reduction in stillbirth rate is higher than ENMR, still the rate is quite high. Early neonatal mortality is more related to the health and care condition at and after delivery while stillbirth has a broader perspective and linked with the maternal condition during pregnancy along with other factors associated at delivery. Evidence suggests that antepartum factors such as prenatal hemorrhage, eclampsia infections, maternal obesity, smoking in pregnancy, and fetal growth restriction are associated with stillbirth [22–24]. At the same time, different intrapartum factors including complications at delivery, time taken to reach the facility, preterm delivery are also associated with stillbirth [25]. Therefore, to increase the rate of reduction of stillbirth focus should also be given on interventions during pregnancy period alongside addressing the factors responsible to ensure safe delivery.

The perinatal mortality rate (PNMR) is a key indicator of overall health condition of a country because of its relationship with quality of health service during pregnancy [1,26]. This is why, many countries with high infant mortality rates had taken major target of reducing perinatal mortality to achieve UN Millennium Development Goal 4 (MDG 4) of reducing under-5 mortality rate [2]. Although global child mortality has been reduced by 53% between 1990 and 2015 [27], the reduction in neonatal mortality has been much slower [28]. The rate of stillbirth was also declined over the past two decades, but many argued that more reduction is possible [29,30]. Therefore, remarkable reduction in child mortality will require interventions focusing on perinatal deaths [28]. Consequently, analysis of trends in PNMR to depict the clear trajectory in the reduction of mortality as well as investigation of the potential risk factors associated with high perinatal mortality would be an advanced step towards formulating effective policies to tackle the issue. The present study therefore attempts to analyze the trends in and determinants of perinatal mortality in Bangladesh using the dataset of four recent Bangladesh Demographic and Health Surveys.

The present study outlined the year-adjusted risk factors such as household, individual and parental-level factors of perinatal mortality in Bangladesh. It was found that perinatal mortality was significantly higher among boys than girls. It is scientifically evident that boys are biologically weaker than girls and more susceptible towards intrauterine growth restriction, prematurity, several diseases and premature death [29,31]. Like other studies of similar nature [32,33], we found that perinatal mortality was significantly higher in rural areas which can be attributed to the remoteness and lack of availabilities of the health service facilities in rural areas. It is important that primary healthcare services in rural areas are equipped with health workers selected from the same communities and resources are to be allocated in an equitable manner [34]. Socioeconomic disparities in perinatal mortality have also been a matter of discourse in low- and middle-income countries (LMICs). Despite considerable global reduction in the perinatal deaths over the last two decades, still neonatal deaths are more pronounced in the poor households [35]. The present study also found that the perinatal mortality was significantly higher in the poor households. This higher rate can be explained by the fact that the deprived people often lack the resources required for essential number of antenatal and postnatal checkups [35,36]. Our study also found that the perinatal deaths were significantly higher among mothers who received less than the recommended four or more antenatal check-ups during their pregnancy period. It was found that the perinatal mortality rate was significantly higher in Sylhet division, which is one of the most hard-to-reach areas of the country. Therefore, initiatives need to be taken for socioeconomically deprived and hard-to-reach areas to reduce the overall PNMR in Bangladesh. Scaling up of interventions like Improving Maternal Neonatal and Child Survival (IMNCS) [37] and Living Goods [38] can be effective in this regard.

We found that unimproved drinking water and sanitation facilities at the households were significantly associated with higher odds of perinatal deaths. These findings are consistent with the findings of the other studies that were carried out in similar settings [39,40]. Therefore, improved water and sanitation facilities should also be ensured alongside delivering emergency obstetric care services at the health facilities. It is also interesting to note that perinatal deaths were significantly higher in households using solid materials for cooking. This is probably resulting due to the higher rate of indoor air population resulting in burning the solid cooking fuel [41,42]. Therefore, focus should also be given on serving the relatively poor households who are more likely to use solid fuel.

The present study also found that higher birth interval was significantly associated with lower perinatal deaths, which is consistent with other studies [43,44]. Higher birth interval generally reduces the risk of the incidence of obstetric complications which might have an effect on perinatal deaths. The present study also pointed that the higher the number of delivered children in the last 5 years is, the higher the odds of perinatal deaths is. This might be due to small birth interval which increases the risk of premature birth or a low birth weight baby. Therefore, small birth interval or taking three or more children within five years period can threaten the health of the child and the mother since these conditions are significantly associated with the higher perinatal deaths.

As found in other studies [44,45], we also found that previous obstetric history of miscarriage or abortion or previous child death increased the odds of perinatal mortality. Several studies [46–48] pointed that maternal obesity increase the risk of abortion, miscarriage and the risk of child mortality. We also found that the odds of perinatal mortality was higher among obese mothers. It was found that the perinatal mortality was significantly higher among mothers aged <20 years. In rural areas of Nepal, Sharma et al. [49] found similar findings as the risk of neonatal deaths was higher among young-aged mothers. In Bangladesh, young mothers are more likely to be from poor households and less educated which may result in poor birth outcome. Maternal employment may have also an adverse effect on child care as the mothers who work outside home might have less time to rest for themselves and breastfeed their children. In our study, we found that the odds of perinatal death was significantly higher among mothers who were working outside home.

We found that the odds of perinatal mortality was significantly higher among mothers with cesarean delivery. Although a study carried out in the USA reported that cesarean deliveries are more prone to be associated with higher perinatal mortality than those of vaginal deliveries many of the cases were complicated cases with higher chances of obstetric complications [50]. In addition to complicated cases being referred for C-section, quality of C-section services, skills of the providers and timeliness of C-section are also known to be risk factors for stillbirths and neonatal mortality in developing country settings [51]. Several studies pointed that perinatal mortality was higher among twins which is probably due to the fact that obstetric complications are higher among mothers having twin babies and often one of the twin babies receives less care due to social customs in Asian and African countries [52–54]. The present study also reported that perinatal deaths were significantly higher among twins compared to the singleton babies. A recent study carried out in Ethiopia [55] found that women’s education and decision making abilities on obstetric care were significantly associated with lower odds of perinatal mortality. The present study also found similar results as women empowerment and level of education were inversely associated with higher odds of perinatal mortality. Moreover, paternal education and occupation had shown significant association with the perinatal mortality in the present study. We found that as the level of paternal education increased, the odds of perinatal mortality reduced. Similar findings were also reported in others studies [55,56]. It was found that the odds of perinatal mortality was significantly lower among children of fathers who were businessmen or service holders. Businessmen or service holders generally earn more which is highly associated with better household socioeconomic conditions and hence seek better health facility which can reduce perinatal deaths.

The study has few limitations. First, investigation of causation from the cross-sectional design is very challenging and hence only associations are truly estimable. Second, both stillbirths and early neonatal deaths were self-reported by mothers and there may arise recall bias. Third, other possible determinants of perinatal mortality such as environmental factors and biological factors of child and parents were not available in the various versions of the BDHS datasets. Fourth, information on pregnancy and delivery complications were also not available for all of the pregnancies. Fifth, pooled datasets were used for the analysis and therefore socioeconomic or other population characteristics may fluctuate over time. However, we adjusted our findings with time and intra-cluster variability. Sixth, as the surveys were not planned to estimate PNMR at the disaggregated level, the estimates of PNMR for different categories need to be interpreted with caution.

Despite these limitations, the study has many strengths. First, the BDHS data are nationally representative and cover all administrative divisions and districts. Second, the average response rates of the BDHS data are very high (98%) such that the selection bias is unlikely to affect the study findings. Third, the BDHS used standardized questionnaires for data collection with a large number of covariates. Fourth, high-quality interviews reduce the possible interview bias while multilayer monitoring system such as regular field visit by survey team, re-interviewing the respondents and spot-checking the completed questionnaire by quality control teams ensure the high quality of data. Fifth, the pooled large data increase the statistical power to explore the trends and potential risk factors over time to generalize the findings to other populations with similar characteristics. This analysis using the pooled BDHS data is quite new. Sixth, we used mixed effect modelling which took into account the naturally hierarchical structure of the data and the variation within clusters, household and individual levels to truly identify the potential risk factors. Seventh, this study provides the clear trajectory of trends and country-specific evidence on risk factors of perinatal deaths to the policymakers and health experts for promoting specific interventions to reduce the high PNMR of the country.

## Conclusion

Our study indicates that although the perinatal mortality rate is declining during the period of 2004–2014, the rate is still quite high in Bangladesh. It is also notable that the reduction rate of stillbirths was comparatively higher than early neonatal deaths which posing the importance of strengthening the emergency obstetric care services during the delivery and postnatal period. It would be beneficial to ensure safe delivery as well as setting up neonatal care centers in all of the healthcare facilities. We identified a number of modifiable risk factors of perinatal mortality such as economic status, maternal BMI, place and type of delivery, mode of delivery etc. This needs to be addressed through undertaking large-scale community based interventions focusing on ensuring proper care during antenatal, delivery and postnatal period.

## Abbreviations

aOR: adjusted odds ratio
BDHS: Bangladesh Demographic and Health Survey
BMI: body mass index
CI: confidence interval
EA: enumeration area
ENMR: early neonatal mortality rate
GEE: generalized estimating equation
PNMR: perinatal mortality rate
OR: odds ratio
WHO: World Health Organization
